# Atlanta Medical and Surgical Union

**Published:** 1884-12

**Authors:** 


### ATLANTA MEDICAL AND SURGICAL UNION.

Reported for THE ATLANTA MEDICAL AND SURGICAL JOURNAL.

STATED MEETING, October 21, 1884.

Dr. W. S. Elkin, President in the Chair.

Dr. W. B. Parks reported a case of double meatus of the penis. He stated that the patient had presented himself for treatment of a gonorrhœa and that when he discovered the abnormality and called the attention of the patient to the fact, he remarked that he did not know anything was wrong, he supposed one place was for the urine and the other for semen. The Doctor stated that the false meatus terminated in a cul-de-sac, about one and a half or two inches deep.

Dr. Parks also reported a case of lacerated perineum which was operated for at once and recovered promptly without difficulty.

Dr. J. D. Wilson reported a case of labor in which there were twins. One child was born in the early morning with both hands extended over the head. The child was dead. The cord was cut and left untied. Some time after dark of the same day, the second child was born dead.

Dr. G. H. Noble asked if fœtal pulsation could be felt in the cord any time after the birth of the first child. Dr. Wilson replied that it could not.

Dr. Noble asked if it would not have been better to have used the forceps and delivered the child earlier. Dr. Wilson thought the result would not have been changed had the forceps been used.

Dr. Noble reported a case of ovariotomy performed for the relief of hemorrhage from the womb, caused by a large interstitial fibroid tumor. He said that he hoped by stopping menstruation the patient would finally recover.

Dr. Wilson asked if the operation was made through the vagina or through the abdominal walls. Dr. Noble replied that the operation was done through the abdomen. He further said that it was best in all cases to operate through the abdomen.

Dr. R. O. Cotter reported a case of blindness as the result of atrophy of the optic nerve that had been relieved almost entirely by the administration of strichnia. The treatment occupied about eight months. He began giving the strichnia in 1-32 grain doses, and increased it to 1-10 grain, three times daily.

Dr. Dyar asked Dr. Cotter if he had used electricity in atrophy of the optic nerve. The Doctor replied that he had, but was not prepared to indorse it.

Dr. D. H. Howell reported a case of an extensive knife wound of the lung, that recovered without suppuration in about ten days. The wound was closed with deep sutures, and the patient kept profoundly under the influence of opium. The pulse never went above 90 per minute.

Dr. E. van Goitsnoven reported a case that he stated puzzled him to some extent. It was either a case of puerperal convulsions, or of strichnia poisoning. He had some very good evidence to lead him to believe that the woman had taken strichnia, and yet there was no evidence that would warrant him in having a legal investigation made. He stated that when he was first called in the patient's pulse was 150. He immediately gave fifteen drops of tincture veratrum, hypodermically. The pulse went down to 60, the convulsions ceased for awhile, then returning with more violence, and patient died in twenty-four hours after the first convulsion.

Dr. Noble asked Dr. van Goitsnoven if he used morphia during the time. He said he thought it would have caused dilatation.

Dr. van Goitsnoven said that he had not, but had used its equivalent—he had used a solution of opium, which he thought filled every indication.

Dr. Wilson said that he had seen a number of cases of puerperal convulsions, in which chloral was used with marked benefit.

Dr. T. D. Love reported a number of cases of puerperal convulsions, and said that he thought morphia in decided doses should always be given.

